# Salvage Liver Transplantation for Recurrent Hepatocellular Carcinoma within UCSF Criteria after Liver Resection

**DOI:** 10.1371/journal.pone.0048932

**Published:** 2012-11-08

**Authors:** Fei Liu, Yonggang Wei, Wentao Wang, Kefei Chen, Lvnan Yan, Tianfu Wen, Jichun Zhao, Mingqing Xu, Bo Li

**Affiliations:** Division of Liver Transplantation, Department of Liver and Vascular Surgery, West China Hospital, Sichuan University, Chengdu, Sichuan Province, China; University of Modena & Reggio Emilia, Italy

## Abstract

**Background:**

Salvage liver transplantation (SLT) is restricted to patients who develop hepatocellular carcinoma (HCC) recurrence within Milan criteria (MC). Little is known about outcomes for SLT in patients with recurrent HCC within University of California San Francisco (UCSF) criteria after liver resection (LR).

**Methods:**

Between January 2001 and December 2011, 380 patients with HCC meeting UCSF criteria, 200 of which were resected (LR group) from a perspective of SLT in case of recurrence, and 180 directly underwent LT (PLT). We compared patient characteristics, perioperative and long-term outcomes between SLT and PLT groups. We also assessed the outcome of LR and PLT groups.

**Results:**

Among the 200 patients in LR group, 86 (43%) developed HCC recurrence and 15/86 (17%) of these patients presented HCC recurrence outside UCSF criteria. Only 39 of the 86 patients underwent SLT, a transplantation rate of 45% of patients with HCC recurrence. Compared with PLT group, LR group showed lower overall survival rate (*P* = 0.005) and higher recurrence rate (*P* = 0.006). Although intraoperative blood loss and required blood transfusion were more frequent in SLT group, the perioperative mortality and posttransplant complications were similar in SLT and PLT groups. The overall survival and recurrence rates did not significantly differ between the two groups. When stratifying by graft type in the SLT group, overall survival and recurrence rates did not significantly differ between deceased donor LT (DDLT) and living donor LT (LDLT) groups. In the subgroup analysis by MC, similar results were observed between patients with recurrent HCC meeting MC and patients with recurrent HCC beyond MC but within UCSF criteria.

**Conclusion:**

Our single institution experience demonstrated that prior hepatectomy and SLT for recurrent HCC within UCSF criteria was feasible and SLT could achieve the same outcome as PLT.

## Introduction

Hepatocellular carcinoma(HCC), which is the fifth most common cancer and the third leading cause of cancer-related death worldwide,is a global health problem [1], [2]. Liver transplantation (LT) is the optimal therapy for patients with HCC and decompensated cirrhosis (Child class B–C) [3] because it removes not only the tumor but also the underlying cirrhotic liver that is at risk for the development of *de novo* HCC. However, the shortage of donor organs represents the major problem in applying primary transplantation to all patients. Liver resection (LR) is still the first-line treatment in patients with HCC and preserved liver function (Child class A) [3], however, the long-term prognosis is undermined by a high incidence of HCC recurrence, up to 50–70% of cases 5 years after surgery [4]–[6]. The combination of both treatments can be a reasonable strategy: HCC patients, within Milan criteria [7] (single nodule ≤5 cm or two or three nodules <3 cm) and with preserved liver function, can successfully undergo LR, limiting the transplantation option to cases of tumor recurrence or hepatic decompensation. LR as a primary therapy with LT in mind for tumor recurrence or deterioration in liver function, so-called salvage transplantation, was first proposed by Majno et al [8].

The two largest initial studies on salvage LT have reported conicting results. Belghiti et al. concluded that liver resection before transplantation does not increase the morbidity or impair long-term survival after LT [9]. Similar results have been reported by Gaudio et al and other workers [10]–[12]. Whereas the other report associated LT after resection with higher operative mortality, an increased risk of recurrence, and a poorer outcome than primary LT [13]. The previous studies on salvage LT were based on deceased donor LT (DDLT); recently, Hwang et al [14] also concluded that combinations of recipient prior hepatectomy and living-donor liver graft were feasible for salvage living donor LT (LDLT), suggesting that salvage procedures should be extended to the living-donor setting. To date, at least 3 reports have analyzed the results of salvage LDLT after liver resection for HCC [14]–[16]. However, few studies have been performed to compare the short and long-term outcomes of LDLT and DDLT in patients with recurrent HCC after LR.

**Figure 1:**
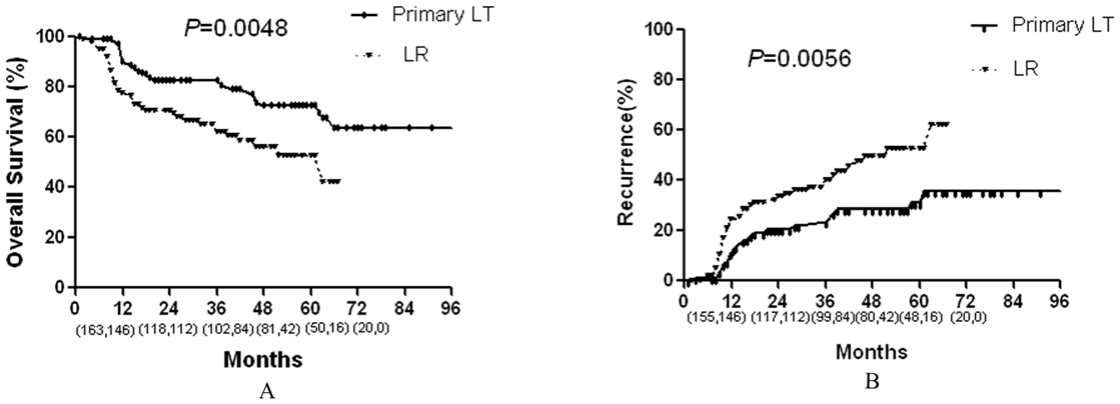
Comparison of the overall survival and recurrence rates between LR and primary LT groups. Numbers in parentheses indicate patients at risk at beginning of each time interval (the front numbers represent primary LT group and the numbers at the back represent the LR group).

Moreover, salvage LT is restricted to patients who develop recurrence within Milan criteria and could represent a loss of opportunity for the subgroup of patients who develop recurrence beyond Milan criteria. Nevertheless, Yao et al [17] proposed that the indication for LT for HCC be expanded to include any solitary tumor less than 6.5 cm, or three or fewer nodules with the largest lesion being less than 4.5 cm and a total tumor diameter of less than 8 cm (the so-called University of California, San Francisco (UCSF) criteria). Similarly, Duffy et al [18] reported their single institution experience with LT for HCC and demonstrated prolonged survival after LT for tumors beyond Milan criteria but within UCSF criteria. Given these reports, we wondered whether the salvage procedure might be useful in patients with recurrent HCC within UCSF criteria after LR.

**Table 1:** Patients and tumor characteristics in the primary and salvage LT groups.

	Types of LT			Types of donor in salvage LT		
	Primary LT	Salvage LT	*P*	DDLT	LDLT	*P*
	N = 180	N = 39	Value	N = 30	N = 9	Value
Gender M/F	162/18	36/3	0.89	28/2	8/1	0.56
Recipient Age	47(26–64)	44(32–65)	0.46	45(32–64)	40(32–57)	0.16
Etiology			0.66			0.56
HBV	172	36		28	8	
Other	8	3		2	1	
Child-Pugh score (A vs. B and C)	36/142	20/19	0.000	16/14	4/5	0.93
MELD score	14.2±5.0	11.0±7.4	0.003	11.5±8.1	9.1±3.6	0.40
Pretransplant treatment			0.97			0.93
TACE	60	10		7	3	
RFA	15	3		2	1	
TACE+RFA	30	5		3	2	
All treatments	105(58.33%)	18(46.15%)	0.17	12	6	0.31
Transplant type			0.91			–
LDLT	40	9		–	–	
DDLT	140	30		–	–	
Serum AFP level, ng/mL			0.02			0.42
≤400	92	28		23	5	
>400	88	11		7	4	
Tumor size (cm)			0.42			0.87
≤5	122	29		23	6	
>5	58	10		7	3	
Tumor number			0.68			0.23
Single	108	22		19	3	
Multiple (2–3)	72	17		11	6	
Microscopic vascular invasion			0.01			0.93
Yes	54	20		16	4	
No	126	19		14	5	
Differentiation			0.08			0.44
Well (n)	42	10		9	1	
Moderate (n)	120	20		14	6	
Poor (n)	18	9		7	2	
Milan criteria			0.08			0.93
Within criteria	122	20		16	4	
Beyond criteria	58	19		14	5	
Satellitosis	50 (27.8%)	9 (23%)	0.55	6 (20%)	3 (33%)	0.70
Follow-up, median with range, (mo)	33 (1–133)	30 (1–82)		34 (1–82)	30 (1–80)	

Abbreviation: M/F, male/female; HBV, hepatitis B virus; AFP, α fetoprotein; TACE, transarterial chemoembolization; RFA, radiofrequency ablation; LT, liver transplantation; LDLT, living donor liver transplantation; DDLT, deceased donor liver transplantation.

Here, we analyzed retrospectively 380 HCC patients within UCSF criteria who underwent LR or LT at our institute between January 2001 and December 2011. We investigated the short- and long-term outcomes of salvage LT for patients with recurrent HCC within UCSF criteria after hepatectomy. In additional, we examined the short- and long-term outcomes of salvage LT for patients by comparing LDLT with DDLT.

**Table 2:** Comparison of operative characteristics and postoperative complications of primary and salvage liver transplantation (LT).

	Types of LT			Types of donor in salvage LT		
	Primary LT	Salvage LT	*P*	Deceased donor	Living donor	*P*
	(n = 180)	(n = 39)	Value	(n = 30)	(n = 9)	Value
Operation time (hour)	9.3±2.0	10.0±1.8	0.06	9.9±1.7	10.6±1.8	0.29
Intraoperative blood loss (ml)	1454±1275	2500±2088	0.00	2270±1544	3267±3346	0.21
Packed RBC transfusion (units)†	5.9±6.1	9.6±9.0	0.007	8.2±6.3	14.2±14.6	0.08
FFP transfusion (units)†	5.2±4.0	5.4±4.1	0.74	4.8±3.7	7.4±5.2	0.11
ICU stay (d), median (range)	10 (3–24)	10 (5–39)	0.32	9 (5–39)	11(6–24)	0.44
Hospital stay (d), median (range)	37 (10–87)	35 (13–86)	0.49	35 (13–82)	36 (14–86)	0.21
Perioperative mortality	8 (4.4%)	2 (5.1%)	1.00	1	1	0.41
Bleeding complication‡	9 (5.0%)	1 (2.6%)	0.81	0	1	0.23
Vascular complication‡	10 (5.6%)	3 (7.7%)	0.89	2	1	0.55
Biliary complication‡	5 (2.8%)	3 (7.7%)	0.31	1	2	0.13
Sepsis	32 (17.8%)	8 (20.5%)	0.69	6	2	1.00
Primary graft dysfunction	4 (2.2%)	0 (0.0%)	1.00	0	0	NA
Acute rejection	12	1	0.54	1	0	1.00

† Including autotransfusions.

‡ Requiring radiologic intervention or reoperation.

RBC, red blood cell; FFP, fresh frozen plasma; ICU, intensive care unit; NA, not applicable.

## Patients and Methods

### Patient Selection

The study was performed from January 2001 to December 2011 and included 380 patients younger than 65 years with HCC within UCSF criteria on imaging. All the 380 patients were potentially transplantable according to UCSF criteria. HCC was diagnosed on the basis of standard clinical criteria, imaging criteria and α fetoprotein levels (AFP), and diagnosis was confirmed by histological examination of the liver specimens. All HCC were examined by experienced hepatopathologists and categorized based on tumor number, size, differentiation grade, microscopic vascular invasion, satellite nodules [19], and fibrosis classification scheme proposed by Ishak et al [20].

**Table 3:** Patient Survival and tumor recurrence.

	Survival (%)			Recurrence (%)		
	1 Yr	3 Yr	5 Yr	1 Yr	3 Yr	5 Yr
LR patients	77	62	52	25	41	53
All LT patients	89	80	69	12	24	32
Primary LT	90	81	72	11	25	31
Salvage LT	88	78	61	14	24	33
Salvage DDLT	92	82	67	14	24	31
Salvage LDLT	87	75	60	13	25	40
Salvage LT within Milan criteria	89	83	66	11	22	29
Salvage LT beyond Milan but within UCSF criteria	88	69	55	17	24	38

LR, liver resection; LT, liver transplantation; LDLT, living donor liver transplantation; DDLT, deceased donor liver transplantation; UCSF, University of California San Francisco; Yr, year.

Among the 380 patients, LR was offered as initial treatment in 200 patients with resectable disease and an adequate estimated post-resection liver function reserve. Anatomic resection, with complete removal of at least one Couinaud’s segment including the tumor area fed by portal branches, was considered [21]. If anatomic resection was not technically possible, we tried to obtain an appropriate margin, greater than 2 cm [22]. Patients with chronic hepatitis B were all treated by appropriate antiviral therapy before and after surgery. Postoperative follow-up included liver function tests, level of serum AFP and abdominal ultrasonography on a 3-month basis in the first 6 months after surgery and on a 6-month basis in the subsequent period, and chest-abdominal CT scan once a year. The strategy was to consider LT for patients who would have developed hepatic HCC recurrence or deterioration of liver function after resection during follow-up. Accordingly, among the 200 transplantable patients, 39 (19.5%) were subsequently transplanted: all for tumor recurrence. All recurrences were discussed at the multidisciplinary meeting and were classified as transplantable or nontransplantable using the same criteria (UCSF criteria).

**Table 4:** Multivariate analysis of factors associated with overall survival for the entire cohort LT patients.

Factor	Hazard ratio	95% CI	p-Value
Microscopic vascular invasion(Yes vs. No)	2.82	1.42–5.62	0.003
Differentiation (poor vs.moderate and well)	6.54	3.42–12.50	<0.001
Satellitosis (Yes vs. No)	1.93	1.03–3.61	0.04

*P* value was obtained by forward stepwise Cox regression model. Initially, 6 clinicopathologic variables (tumor number, tumor size, microscopic vascular invasion, differentiation, satellitosis, Milan criteria) were included in this model, and finally three factors remained as a significant variable.

Patients with deteriorated liver function or unresectable disease were evaluated for LT. The 180 patients transplanted for HCC in the study period were selected according to the following pretransplant criteria: age <65 years, absence of metastatic lymph nodes or extrahepatic spread at the preoperative evaluation, absence of macroscopic vascular invasion, no history of other malignant tumors within the last 5 years, HCC meeting UCSF criteria. Recurrence was defined as the appearance of a new lesion with features of HCC on imaging. In our institution, the criteria for salvage LT were basically similar to those for primary LT. Preoperative staging routinely included hepatic ultrasound, chest and abdominal computer tomography (CT), and bone scintigraphy to look for any extrahepatic tumor spread. In order to avoid the progression of the tumor in the waiting list period, pretreatment for HCC that included transarterial chemoembolization (TACE), radiofrequency ablation (RFA), and a combination of these strategies had been adopted.

**Figure 2:**
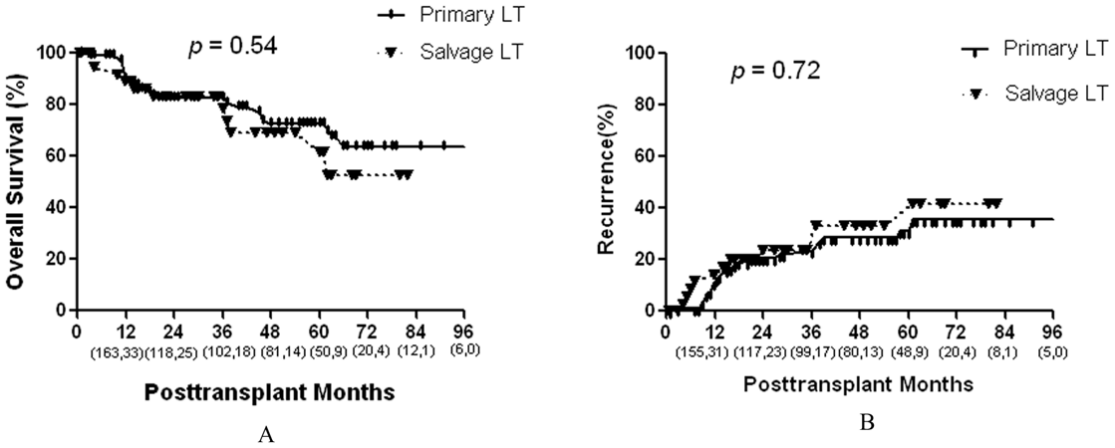
Comparison of the overall survival and recurrence rates between primary and salvage LT groups. Numbers in parentheses indicate patients at risk at beginning of each time interval (the front numbers represent primary LT group and the numbers at the back represent the salvage LT group).

A total of 219 patients underwent primary or salvage LT for HCC within UCSF criteria. All the liver grafts were from brain dead donors or living donors. The selection criteria for the donor and recipient as well as surgical techniques for both donor and recipient operations have been described in detail elsewhere [23], [24]. A detail description of the immunosuppression regimen was described in previous investigation [25]. All patients were followed after surgery by our surgical team, with CT scans of the chest and abdomen every 3 months for the first 2 years and every 6 months thereafter. Also, AFP level was measured every 3 months. Additional imaging techniques (magnatic resonance imaging (MRI), bone scintigraphy) were performed if recurrent HCC was suspected. The patients were followed until April 2012 or their death.

**Figure 3:**
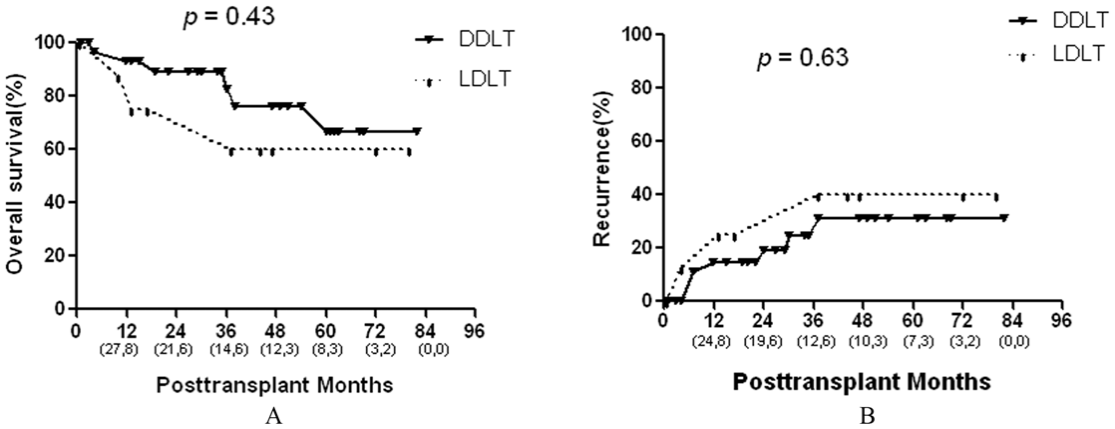
Comparison of the overall survival and recurrence rates between DDLT and LDLT in the salvage LT group. Numbers in parentheses indicate patients at risk at beginning of each time interval (the front numbers represent salvage DDLT group and the numbers at the back represent the salvage LDLT group).

### Ethics Statement

All clinical investigations were in accordance with the ethical guidelines of the Declaration of Helsinki. Ethical approval was obtained from the Committee of Ethics in West China Hospital of Sichuan University. Living and deceased donations were voluntary and altruistic in all cases, and written informed consent was obtained from both donors and recipients before surgery.

**Figure 4:**
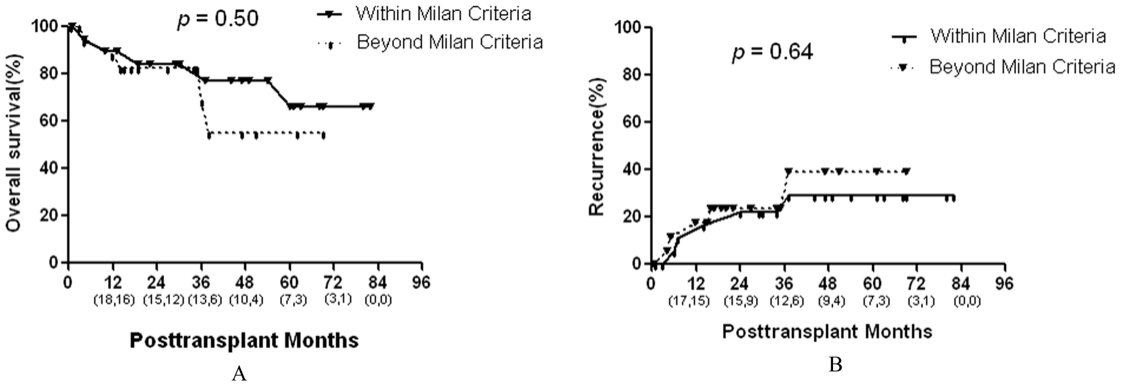
Comparison of the overall survival and recurrence rates in the salvage LT group according to the Milan criteria. Numbers in parentheses indicate patients at risk at beginning of each time interval (the front numbers represent salvage LT for HCC within Milan criteria and the numbers at the back represent the salvage LT for HCC beyond Milan but within UCSF criteria).

### Design of the Study

LR in patients potentially eligible for transplantation (n = 200) was compared with primary LT patients (n = 180), to assess the outcome of each treatment strategy. Survival in each group was calculated from the time of the primary procedure (LR or LT). Salvage LT after LR for HCC (n = 39) was compared to primary LT for HCC (n = 180) to assess the operative risk and the postoperative complications of this surgical procedure. Moreover, we performed subgroup analysis in the salvage LT group by graft type (LDLT or DDLT) and selection criteria (within Milan criteria or beyond Milan but within UCSF criteria). Survival in each group was calculated from the time of transplantation.

### Statistical Analysis

Patient baseline characteristics are expressed as mean ± standard deviation (SD) and median with range for continuous data, and as frequency for categorical data. Statistical analysis was performed using Student *t* test, Mann-Whitney test, *x^2^* test, Fisher’s exact test, log-rank test, and Cox regression. Cumulative overall survival and recurrence rates were calculated using the Kaplan-Meier method, and the differences between curves were evaluated using the log-rank test. The Cox model with the determination of the hazard ratio was applied to evaluate the risk associated with prognostic variables. Univariate results were reported as hazard ratios with 95% confidence intervals. The variables reaching statistical significance by univariate analysis were then included for multivariate analysis with proportional hazard regression. *P*<0.05 was considered statistically significant. All statistical analyses were performed using SPSS version 16.0 for Windows statistical software (SPSS Inc., Chicago, IL, USA).

## Results

### LR Versus Primary LT

#### Demographic data and tumor characteristics

The clinical and tumor characteristics of patients are listed in Table S1. Child-Pugh class-A patients predominated in the LR group and class-B and -C patients in the LT group. Also, the MELD score was likewise significantly lower in the LR group than in the LT group (*P* = .001).Preoperative nonsurgical treatments (TACE, RFA) were more prevalent in the primary LT group (*P*<.001). Moreover, a single tumor was more common in the LR group than in the LT group. No significant differences were observed regarding other clinical and tumor characteristics between LR group and LT group.

#### HCC recurrence in LR group and primary LT group

Among 200 patients eligible for transplantation that underwent LR, 86/200 (43%) developed HCC recurrence and 15/86 (17%) of this subgroup of patients presented an HCC recurrence outside UCSF criteria. The most common site of recurrence was intrahepatic, and only ten cases of HCC recurrence was extrahepatic (including lung, right adrenal gland, bone).The median time to recurrence was 12.9±12 months (range, 6–67) with no significant difference between recurrence within UCSF and beyond UCSF (12.5±12 versus 13.2±11 months, *P* = NS). Among the 71 patients with HCC recurrence within UCSF, 39 (54.9%) underwent a salvage LT with a median time on the waiting list of 2 months. The remaining 32 patients with HCC recurrence within UCSF criteria were not transplanted for the following reasons: in 10 cases because they were over 65 years at the time of HCC recurrence, in 5 cases due to death on the waiting list, the occurrence of significant comorbidities during follow-up representing contraindications for LT (severe ischemic heart disease) in 5 patients, and 12 patients were denied LT at the time of recurrence (n = 5) or were lost to follow-up (n = 7).

Among 180 patients underwent primary LT, 49/180 (27.2%) developed HCC recurrence and 6/49 (12%) of this subgroup of patients presented an HCC recurrence outside UCSF criteria. The most common site of recurrence was intrahepatic, and only four cases of HCC recurrence was extrahepatic (including lung and lumbar vertebra). The median time to recurrence was 20±14 months (range, 9–61). The mean time to recurrence in the primary LT group was significantly longer than that in the LR group (*P* = 0.038).

#### Long-Term outcomes

Cumulative overall survival and recurrence curves of the two groups are shown in Fig 1A and Fig 1B. LR patients had 1-, 3- and 5-year survival rates of 77, 62, and 52%, respectively, versus 90, 81, and 72% in the primary LT group, respectively. This trend in improved survival with primary LT was statistically significant (*P* = .005). Meanwhile, LR patients had 1-, 3- and 5-year recurrence rates of 25, 41, and 53%, respectively, versus 11, 25, and 31% in the primary LT group, respectively. This trend in increased recurrence with LR was statistically significant (*P* = .006).

### Salvage LT Versus Primary LT

#### Demographic data

The demographic data and tumor characteristics of LT patients are presented in Table 1. No significant differences were observed regarding age, sex, or liver disease etiology between primary LT group and salvage LT group. The majority of HCCs in both groups were related to hepatitis B virus (HBV) infection. However, the proportion of Child-Pugh classification A was significantly higher in the salvage LT group than in the primary LT group (*P*<.001). The MELD score was likewise significantly lower in the salvage LT group than in the primary LT group (*P* = .003). Fifty-eight percent of patients had antitumor treatment before LT in the primary LT group, including TACE, RFA, and a combination of the two strategies; while 46% in the salvage LT group. The median interval on the waiting list was 3 months (range 1–10 months) for patients who underwent primary LT, 2 months (range 1–7 months) for patients who underwent salvage LT.

Among the 39 salvage LT patients, 10 patients underwent major hepatectomy before LT and the remained 29 patients underwent minor liver resection. Graft types included cadaveric whole organs in 30 patients (salvage DDLT group) and right lobe living donor grafts (adult to adult) in 9 patients(salvage LDLT group). Age, sex, liver disease etiology, Child-Pugh score, MELD score, and pretransplant treatments did not significantly differ between the two groups.

#### Tumor characteristics

There was no significant difference in neoplasm size, tumor number, differentiation or satellitosis between the primary and salvage LT groups. Furthermore, no difference in Milan criteria was apparent. However, patients in the salvage LT group showed a greater incidence of microscopic vascular invasion (*P* = .01). Moreover, a higher proportion of patients in the salvage LT group had the preoperative serum AFP level less than 400 ng/mL (*P* = .02). Similar results were observed between salvage DDLT and salvage LDLT groups, except for the microscopic vascular invasion and preoperative serum AFP level. Patients in the salvage LDLT group had a greater proportion of multiple tumors than those in the salvage DDLT group (66.7% vs. 36.7%); however, this difference was not statistically significant.

#### Perioperative outcomes

Primary LT versus Salvage LT: Operation profiles and posttransplant complications are summarized in Table 2. Operation time was not greatly prolonged in the salvage compared with the primary LT group. However, patients in the salvage LT group showed significantly more intraoperative blood loss (*P*<.001) and required more packed red blood cell transfusion (*P* = .007). The incidence rates of various posttransplant complications were similar in the salvage and primary LT groups. No difference was observed in perioperative mortality, intense care unit (ICU) stay or hospital stay duration.

Salvage DDLT versus Salvage LDLT: No significant differences were observed between the two groups regarding operation time, intraoperative blood loss, postoperative complications, including bleeding, vascular complication, biliary complication, sepsis, primary graft dysfunction and acute rejection. Patients in the salvage LDLT group required more packed red blood cell transfusion, however, this difference was not statistically significant (*P* = .08). Similarly, no difference was observed in perioperative mortality, ICU stay or hospital stay duration between salvage DDLT group and salvage LDLT group.

#### Long-Term outcomes

Survival and tumor recurrence data are shown in Table 3. Overall survival for the entire cohort LT patients at 1, 3, and 5 years after transplantation were 89%, 80%, and 69%; and the tumor recurrence rates for the entire cohort LT patients at 1, 3, and 5 years after transplantation were 12%, 24%, and 32% respectively.

The factors related to survival in the cohort LT patients are presented in Table S2. Univariate analysis showed that tumor number, tumor size, microscopic vascular invasion, poor differentiation, satellitosis nodules, and tumor beyond Milan criteria were significantly associated with reduced survival after LT for HCC. On multivariate analysis (Table 4), only microscopic vascular invasion (*P* = .003), poor differentiation (*P* <.001), and satellitosis nodules (*P* = .04 ) independently predicted poor survival; tumor number, tumor size, and tumor beyond Milan criteria did not independently inuence post-transplant survival. To specially mention, the treatment modality (primary vs. salvage LT) did not affect overall survival significantly on multivariate analysis.

Primary LT versus Salvage LT: Cumulative overall survival curves of the two groups are shown in Fig 2A. Patients in the primary LT group had slightly higher 5-year survival rates compared with patients in the salvage LT group (5-year rates, 72% vs. 61%, respectively); however, this difference was not statistically significant (*P* = .54). The 5-year recurrence rates also did not significantly differ between the two groups (31% vs. 33%, respectively, Fig 2B).

Moreover, we divided the results obtained in two periods: patients enrolled from 2001 to 2006 and after 2006. Survival and tumor recurrence data during the two periods are shown in Table S3. From 2001 to 2006, 68 HCC patients within UCSF criteria underwent primary LT, while 17 patients with recurrent HCC meeting the criteria underwent salvage LT. The long-term overall survival and recurrence rates did not differ significantly between primary LT and salvage LT groups during the first period (overall survival, *P* = .46; recurrence, *P* = .62). From 2006 to 2011, 112 HCC patients within UCSF criteria underwent primary LT, while 22 patients with recurrent HCC meeting the criteria underwent salvage LT. The long-term overall survival and recurrence rates did not differ significantly between primary LT and salvage LT groups during the second period (overall survival, *P* = .20; recurrence, *P* = .55).

Salvage DDLT versus Salvage LDLT: Long-term overall survival rates did not differ significantly between salvage DDLT and salvage LDLT groups (5-year rates, 67% and 60%, respectively, Fig 3A). The 5-year recurrence rates also did not significantly differ between the two groups (31% vs. 40%, respectively, Fig 3B).

Long-term outcomes in the salvage LT group according to selection criteria: The 39 patients who underwent salvage LT were divided into two groups (group A, recurrent HCC within Milan criteria; group B, recurrent HCC beyond Milan but within UCSF criteria). Patients with recurrent HCC within Milan criteria had slightly higher 3-year and 5-year survival rates compared with those with recurrent HCC beyond Milan but within UCSF criteria (3-year rates, 83% vs. 69%; 5-year rates, 66% vs. 55%, respectively, Fig 4A); however, this difference was not statistically significant (*P* = .50). The 1, 3, and 5-year recurrence rates also did not significantly differ between the two groups (Fig 4B).

## Discussion

In this study, we retrospectively evaluated the outcome of patients with HCC meeting UCSF criteria selected for LR and LT. The overall survival after LR in our series was 52% at 5 years. However, Facciuto et al [11] reported that 5-year survivals of 35% for HCC patients beyond Milan criteria, and Fong et al. [4], reported 3-year and 5-year survivals of 48% and 33%, respectively, for patients with tumors greater than 5 cm. One explanation for this discrepancy is that a large proportion of HCC in our series were within Milan criteria. The 1-, 3- and 5-year survival rates were 90, 81, and 72% and 1-, 3- and 5-year recurrence rates of 11, 25, and 31% in the primary LT group, which showed reduced incidence of recurrence and improved survival compared to LR group. In fact, similar results had been observed by some previous studies [26]–[28]. However, the choice of a particular treatment option would depend on individual liver function and availability of a donor liver in the setting of shortage of donor organs. Our purpose was not to compare two treatment groups, but to ascertain the outcome of a combined strategy employing prior LR and salvage LT for HCC meeting UCSF criteria.

Moreover, LR has several advantages for patients with HCC meeting UCSF criteria and well-preserved liver function. First, LR is technically far less complex than LT and can be performed without delay. Second, for patients with HCC meeting UCSF criteria and well-preserved liver function, LR would achieve recurrence-free, long-term survival in nearly a half of patients in our series, and a half of grafts are saved for the community and can be transplanted to other patients who have no other alternative. Last but not least, patients with HCC beyond Milan criteria are at higher risk of disease progression and higher dropout rate on waiting list [29]. LR could achieve initial control of the tumor and decrease dropout rate, leaving salvage LT as a reserve option to manage recurrence.

The Milan criteria [7] for HCC have been widely used as the guideline for the selection of candidates for LT in many transplantation centers. In recent years, some investigators have argued that the Milan criteria are too restrictive and limit the transplant option at a time when the incidence of HCC is increasing. Several recent series have demonstrated good outcomes using expanded criteria (the UCSF criteria), with long-term survival similar to Milan criteria [18], [30]–[32]. Similar results were observed in our study. In the present report of 219 patients managed in a single institution, LT is confirmed as appropriate and effective treatment for patients with HCC meeting UCSF criteria, with 1, 3, 5-year survival rates of 89%, 80%, 69% and 1, 3, 5-year recurrence rates of 12%, 24%, 32%, respectively.

Salvage LT, which was first proposed by Majno et al [8], is restricted to patients who develop recurrence within Milan criteria and could represent a loss of opportunity for the subgroup of patients who develop recurrence beyond Milan criteria. Recently, Kaido et al [15] reported that the selection criteria for salvage LT to treat recurrent HCC could be expanded to Kyoto criteria (tumor number ≤10, the maximal diameter of each tumor was ≤5 cm and serum des-gamma-carboxy prothrombin levels of ≤400 mAU/mL). However, the patient cohort in their study was small and the Kyoto criteria was based on salvage LDLT. As mentioned above, LT for HCC within UCSF criteria could achieve excellent long-term survival similar to Milan criteria. Nevertheless, little is known about outcomes for salvage LT in patients with recurrent HCC within UCSF criteria after LR. Hence, a study about salvage LT for recurrent HCC within UCSF criteria was of great value. Here, we found favorable short- and long-term outcomes in 39 patients who underwent LT for recurrent HCC meeting UCSF criteria after LR.

Although the intraoperative blood loss and required blood transfusion were more frequent than primary LT group, the operation time was not prolonged in salvage LT group. Moreover, the perioperative mortality and posttransplant complications were similar in the salvage and primary LT groups. The larger volume of intraoperative blood loss in the salvage LT group might be caused by intra-abdominal adhesion. Heavy adhesions are often encountered after prior LR, and minute collaterals penetrate into such adhesions in patients with portal hypertension. Inattentive dissection of such perihepatic adhesions could result in many uncontrollable sites of pinpoint bleeding at the dissection surface. The similar phenomenon was also observed by Adam et al and Hwang et al [13.14]. Adam et al [13] reported that their 17 patients who underwent salvage LT after LR versus 195 who underwent primary LT for HCC showed higher blood requirements. Hwang et al [14] reported that bleeding complications occurred more frequently in patients undergoing salvage LDLT than those undergoing primary LDLT.

The overall survival and recurrence rates did not significantly differ between the primary LT and salvage LT groups. However, both in primary LT and in salvage LT groups, the rates of HCC recurrence were higher compared to previous literature reports [8]–[10], [13]–[15]. For example, the 5-year recurrence rates in the primary LT and salvage LT groups were 31% and 33% in the present study. Nevertheless, Kaido et al [15] reported that the 5-year recurrence rates in the primary LT and salvage LT groups were 8% and 22%. These inconsistent results may be due to different criteria for salvage LT. Such as, most previous studies used Milan criteria to screen patient for salvage LT; while the UCSF criteria was used to screen patient for salvage LT in our study. Other factors in these studies such as small sample size and different ethnicities could also cause the inconsistent results. When stratifying by Milan criteria in the salvage LT group, the overall survival and recurrence rates did not significantly differ between patients with recurrent HCC meeting Milan criteria and patients with recurrent HCC beyond Milan but within UCSF criteria, which indicated that salvage LT for recurrent HCC beyond Milan but within UCSF criteria was feasible.

The initial studies on salvage LT were based on DDLT; however, several recent series [14]–[16] indicated that combinations of recipient prior hepatectomy and living-donor liver graft were feasible for salvage LDLT. However, to the best of our knowledge, few studies have been performed to compare the short- and long-term outcomes of LDLT and DDLT in patients with recurrent HCC after LR. In the primary LT setting, Gondolesi et al. [33] reported comparable results in HCC patients who underwent LT using living donors or deceased donors. Di Sandro et al [34] also reported that LDLT guarantees the same long-term results as DDLT. In the present study, no significant differences were observed between salvage DDLT and LDLT group regarding operation time, intraoperative blood loss, postoperative complications and perioperative mortality. Patients in the salvage LDLT group required more packed red blood cell transfusion, however, this difference was not statistically significant. Moreover, the long-term overall survival and recurrence rates did not differ significantly between salvage DDLT and salvage LDLT groups, which indicated DDLT and LDLT could equally achieve the salvage procedure.

The most common cause of death in patients who underwent either LR or LT was HCC recurrence. The pathologic factors associated with biologic aggressiveness include tumor differentiation grade histologic type, presence of a peritumoral capsule, and microscopic vascular invasion [35]–[37]. Not surprisingly, microscopic vascular invasion, poor differentiation, and satellitosis nodules were independent predictors of poor survival in our series. These determinants have been associated with poor outcome in prior series [37]–[39]. The univariate analysis showed that tumor size, tumor number, and tumor beyond Milan criteria were also significantly associated with reduced survival after LT for HCC, however, these factors were excluded in the multivariate analysis. One explanation for the phenomenon is that these preoperative tumor characteristics may not be better indicators of post-LT tumor biology behavior. Based on personal observations, we found that some patients with small tumors, however, will still do poorly after transplant while others outside UCSF boundaries can still surprise us and do well. The multinational database analysis from Onaca also showed good results for some expanded tumors, with 5-year survival above 60% for patients with 2 to 4 tumors from 3 to 5 cm [30].Some tumors, even large or extensive ones, exhibit less aggressive biology than do others.

Preoperative locoregional therapy, which was used in more than half of our patients, was not associated with improved post-transplant survival on multivariate analysis. Our results were consistent with some previous reports [17], [29]. Locoregional treatments do, however, have the potential to prevent waiting list drop out due to tumor progression. Lu et al [40] reported RFA to be an effective bridge to LT, as it limited the dropout rate from LT candidacy to only 5.8% and contributed to post-LT survival rates of 85% and 76% at 1 and 3 years after transplant. Hence; locoregional therapy remains a viable tool for local tumor control, particularly in patients with advanced HCC facing prolonged waits for LT.

In conclusion, salvage LT for recurrent HCC within UCSF criteria was feasible and it could achieve the same outcome as primary LT. Moreover, salvage LDLT could achieve the same short- and long-term outcomes as salvage DDLT. Since our study was limited to a single center experience and a retrospective research, it is critical that multicentric studies and an intention-to-treat analysis should be performed to confirm our results.

## Supporting Information

Table S1 **Patients and tumor characteristics in the primary LR and LT groups.**
(DOC)

Table S2 **Univariate analysis of factors associated with overall survival for the entire cohort LT patients.**
(DOC).

Table S3 **Evaluate the overall survival and tumor recurrence rates for primary LT and salvage LT groups by dividing the results obtained in two periods.**
(DOC).
