# Supplementary material for: Salvage Liver Transplantation for Recurrent Hepatocellular Carcinoma within UCSF Criteria after Liver Resection
Source: PLoS One. 2012 Nov 8;7(11):e48932. doi: 10.1371/journal.pone.0048932 (PMC3493590; doi:10.1371/journal.pone.0048932)
Supplement: Table S1 — Patients and tumor characteristics in the primary LR and LT groups. (DOC) [file pone.0048932.s001.doc]

**Table S1.** Patients and tumor characteristics in the primary LR and LT groups

|  | Types of treatments | | |
| --- | --- | --- | --- |
|  | Primary LT | LR^a^ | *P* |
|  | N=180 | N=200 | Value |
| Gender M/F | 162/18 | 175/25 | 0.44 |
| Age | 47(26-64) | 45(28-60) | 0.58 |
| Etiology |  |  | 0.21 |
| HBV | 172 | 185 |  |
| Other | 8 | 15 |  |
| Child-Pugh score (A vs. B and C) | 36/142 | 145/55 | 0.000 |
| MELD score | 14.2±5.0 | 8.0±4.4 | 0.001 |
| Pretransplant treatment |  |  | 0.000 |
| TACE | 60 | 35 |  |
| RFA | 15 | 5 |  |
| TACE+RFA | 30 | − |  |
| Serum AFP level, ng/mL |  |  | 0.23 |
| ≤ 400 | 92 | 90 |  |
| ＞400 | 88 | 110 |  |
| Tumor size (cm) |  |  | 0.80 |
| ≤5 | 122 | 138 |  |
| ＞5 | 58 | 62 |  |
| Tumor number |  |  | 0.000 |
| Single | 108 | 155 |  |
| Multiple (2-3) | 72 | 45 |  |
| Microscopic vascular invasion |  |  | 0.22 |
| Yes | 54 | 72 |  |
| No | 126 | 128 |  |
| Differentiation |  |  | 0.20 |
| Well (n) | 42 | 36 |  |
| Moderate (n) | 120 | 134 |  |
| Poor (n) | 18 | 30 |  |
| Milan criteria |  |  | 0.80 |
| Within criteria | 122 | 138 |  |
| Beyond criteria | 58 | 62 |  |
| Satellitosis | 50 (27.8%) | 63 (31.5%) | 0.43 |
| Follow-up, median with range, (mo) | 33 (1-133) | 22 (1-67) | 0.033 |

Abbreviation: M/F, male/female; HBV, hepatitis B virus; AFP, α fetoprotein; TACE, transarterial chemoembolization; RFA, radiofrequency ablation; LT, liver transplantation; LR, liver resection.

a, LR potentially transplantable according to University of California San Francisco criteria.
