# Supplementary material for: Salvage Liver Transplantation for Recurrent Hepatocellular Carcinoma within UCSF Criteria after Liver Resection
Source: PLoS One. 2012 Nov 8;7(11):e48932. doi: 10.1371/journal.pone.0048932 (PMC3493590; doi:10.1371/journal.pone.0048932)
Supplement: Table S2 — Univariate analysis of factors associated with overall survival for the entire cohort LT patients. (DOC). [file pone.0048932.s002.doc]

Table S2. Univariate analysis of factors associated with overall survival for the entire cohort LT patients.

| Factor | Hazard ratio | 95% CI | p-Value |
| --- | --- | --- | --- |
| Age | 0.98 | 0.95–1.01 | 0.23 |
| Male gender | 1.22 | 0.73–2.04 | 0.45 |
| Tumor number (multiple vs. single ) | 2.77 | 1.33–5.77 | 0.007 |
| Tumor size (＞5cm vs. ≤ 5cm) | 2.64 | 1.46–4.79 | 0.001 |
| Microscopic vascular invasion  (Yes vs. No) | 4.34 | 2.23–8.43 | ＜0.001 |
| Differentiation (poor vs. moderate and well) | 9.12 | 4.94–16.83 | ＜0.001 |
| Satellitosis (Yes vs. No) | 3.04 | 1.67–5.53 | ＜0.001 |
| Milan criteria (Beyond vs. Within) | 2.07 | 1.15–3.73 | 0.02 |
| Serum AFP level (＞400 ng/mL vs. ≤400 ng/mL) | 1.67 | 0.93–3.00 | 0.09 |
| MELD score | 0.98 | 0.93–1.04 | 0.56 |
| Child-Pugh score ( A vs.B and C) | 0.98 | 0.82–1.16 | 0.80 |
| Etiology (HBV vs. Other) | 0.80 | 0.25–2.57 | 0.70 |
| Pretransplant treatment (With vs. without) | 1.74 | 0.84–3.62 | 0.14 |
| Treatment (Primary LT vs. Salvage LT) | 0.66 | 0.36–1.21 | 0.18 |
