# Supplementary material for: Salvage Liver Transplantation for Recurrent Hepatocellular Carcinoma within UCSF Criteria after Liver Resection
Source: PLoS One. 2012 Nov 8;7(11):e48932. doi: 10.1371/journal.pone.0048932 (PMC3493590; doi:10.1371/journal.pone.0048932)
Supplement: Table S3 — Evaluate the overall survival and tumor recurrence rates for primary LT and salvage LT groups by dividing the results obtained in two periods. (DOC). [file pone.0048932.s003.doc]

Table S3. Evaluate the overall survival and tumor recurrence rates for primary LT and salvage LT groups by dividing the results obtained in two periods.

|  | Survival (%) | | | Recurrence (%) | | |
| --- | --- | --- | --- | --- | --- | --- |
|  | 1 Yr | 3 Yr | 5 Yr | 1Yr | 3 Yr | 5Yr |
| From 2001 to 2006 | | | | | | |
| Primary LT | 89 | 81 | 67 | 12 | 26 | 31 |
| Salvage LT | 86 | 72 | 60 | 15 | 27 | 34 |
| From 2006 to 2011 | | | | | | |
| Primary LT | 91 | 83 | 78 | 10 | 19 | 26 |
| Salvage LT | 88 | 81 | 65 | 13 | 21 | 31 |

LT, liver transplantation, Yr, year.
